# Artificial intelligent-driven decision-making for automating root fracture detection in periapical radiographs

**DOI:** 10.1038/s41405-024-00260-1

**Published:** 2024-10-01

**Authors:** Riem Abdelazim, Eman M. Fouad

**Affiliations:** 1 Department of Information Systems, Faculty of Information Technology, Misr University for Science and Technology, Giza, Egypt; 2 Division of Endodontics, Faculty of Oral and Dental Surgery, Misr University for Science and Technology, Giza, Egypt

**Keywords:** Endodontics, Periapical radiographs

## Abstract

**Background:**

The detection and early diagnosis of root fractures can be challenging; this difficulty applies particularly to newly qualified dentists. Aside from clinical examination, diagnosis often requires radiographic assessment. Nonetheless, human fallibility can introduce errors due to a lack of experience.

**Aim:**

The proposed system aimed to assist in detecting root fractures through the integration of artificial intelligence techniques into the diagnosis process as a step for automating dental diagnosis and decision-making processes.

**Materials and method:**

A total of 400 radiographic images of fractured and unfractured teeth were obtained for the present research. Data handling techniques were implemented to balance the distribution of the samples. The AI-based system used the voting technique for five different pretrained models namely, VGG16, VGG19, ResNet50. DenseNet121, and DenseNet169 to perform the analysis. The parameters used for the analysis of the models are loss and accuracy curves.

**Results:**

VGG16 exhibited notable success with low training and validation losses (0.09% and 0.18%, respectively), high specificity, sensitivity, and positive predictive value (PPV). VGG19 showed potential overfitting concerns, while ResNet50 displayed progress in minimizing loss but exhibited bias toward unfractured cases. DenseNet121 effectively addressed overfitting and noise issues, achieving balanced metrics and impressive PPVs for both fractured and unfractured cases (0.933 and 0.898 respectively). With increased depth, DenseNet169 demonstrated enhanced generalization capability.

**Conclusion:**

The proposed AI- based system demonstrated high precision and sensitivity for detecting root fractures in endodontically treated teeth by utilizing the voting method.

## Background

Early and accurate detection of root fractures is critical, given its implications for treatment modalities and prognosis. The predisposing factors that underlie the treatment options have been highlighted in the literature, with the emphasis of accurate and early detection on prognosis [1]. The prevalence of root fractures can reach up to 7%, and the underlying etiological classification includes both traumatic and chronic fatigue root fractures [2]. In the pursuit of diagnosing such fractures, the detection of root fractures involves radiographic assessment which offers invaluable insights together with evidence from clinical assessments. However, they are considered as subjective processes affected by the degree of examiner´s experience [3]. Moreover, less experienced operators may inadvertently misdiagnose dental root fractures due to a lack of exposure to a diverse range of cases and diagnostic nuances.

Three-dimensional (3D) dental imaging modalities, such as cone beam computed tomography (CBCT) could enable clinicians to visualize dental structures in multiple planes with exceptional clarity and high accuracy for detecting root fractures. This is particularly true due to the limitations of two- dimensional (2D) radiographic images, such as distortion, inherent superimposition of structures and the inability to visualize fractures in multiple planes which can lead to false negatives or delays in diagnosis [4]. However, limitations like image artifact, cost, and radiation exposure argue against its routine implementation in daily routine [5]. The periapical radiograph is the standard radiographic modality in dental clinics and the first aid for screening [6].

Evolving technology has changed the whole interface of diagnostic methodology as well as the degree of precision of restorative and endodontic treatment modalities [7]. Artificial intelligence (AI) technology has been intensely introduced into almost every branch of dentistry and is involved in diagnostic aspects and decision making [8]. In the context of dentistry, the integration of AI into information systems (IS) has shown promising potential [9]. The literature provides evidence where AI algorithms support in the interpretation of dental imagery, diagnosis of oral conditions, and prediction of potential issues based on input data from X-rays, patient records, or other dental imaging modalities [10, 11].

Incorporating AI can offer a rapid means of predicting the likelihood of specific case occurrences [12]. The challenge of accurately diagnosing root fractures, even among dental experts, is highlighted in the literature [13]. An extended utilization of such a system, where dentists seek to ensure the accuracy of their diagnoses, can also be forwarded to patients seeking a second medical opinion on their case in a quest to reinforce the reliability of their medical assessments.

Convolutional Neural Networks (CNNs) is a subclass of neural networks that can efficiently identify and classify dental structures, detect anomalies like cavities, root fractures, and periodontal diseases, and assist in the accurate segmentation of teeth and surrounding anatomical features, consequently advancing image-based diagnosis [13].

The early attempts involved the validation of a probabilistic neural network design for detection of vertical root fractures in periapical radiographic images and pointed out its potential for dental fracture detection [3]. A study held by Johari et al. demonstrated that the neural network performs more effectively with CBCT images compared to periapical radiographs for both endodontically and non-endodontically treated single rooted premolars [14]. In a comparable effort, Hu et al. deployed AI algorithms, including ResNet50, DenseNet169, and VGG19, to detect fractures in a dataset of CBCT images comprising 276 teeth. Their findings significantly favored the ResNet50 algorithm over the other AI models and even outperformed domain experts [15].

This research article builds on previous work published as an e-Poster [16]. However, independently training each AI model did not achieve the highest accuracy or sensitivity. Herein, the present study aims to validate five AI algorithms, comparing their performances regarding specificity, sensitivity, and positive predictive value (PPV) in detecting root fractures. Moreover, introducing a new application of a voting mechanism in this context. to reduce human errors and improve the overall quality of patient care.

## Materials and methods

### Sample preparation

The study at hand was reviewed and the ethical approval was obtained from the Institutional Review Board of Misr University for Science and Technology (MUST IRB) under approval number 2022/0097. Single rooted anterior teeth, extracted for periodontal reasons, were selected for the present study. Both extracted endodontically treated maxillary and mandibular teeth were included. The 400 collected teeth were divided into two main groups of equal size: fractured and intact roots. The number of root samples was based on sample size calculation using Epicalc program version 1.02 assuming a power of 95% and alpha = 0.05, with reference to previous research [14]. Root fracture was artificially created in the horizontal plane to simulate incomplete root fractures, with the aid of a diamond cutting disc (#2, 0.15 × 22 mm). All teeth were radiographed with digital radiographic image plate (photostimulable storage phosphor plate) size 2 of the VistaScan system (Dürr Dental AG, Beitigheim-Bissingen, Germany) and processed digitally through DBSWIN software.

### Data preparation

#### Processing configuration

The radiographic images constituted the dataset under study. This set of images was configured for testing using the five identified AI models, namely: VGG16, VGG19, ResNet50, DenseNet121, and DenseNet169. Data handling constituted a balanced distribution of both fractured and unfractured, which guards against potential bias and stresses transparency in the research process. The software used for graph generation was Matplotlib using the Python library. For this research, a standardized process of the configurations across the five models was implemented. These configurations were carefully selected to promote consistent and effective training across all the models, ensuring robustness and facilitating accurate root fracture detection.

The basic configuration of this dataset was deployed, such as the number of epochs fixed at 50 epochs for consistent training. Moreover, the study employed regularization techniques in the form of dropout with a value of 0.3 to mitigate overfitting during training. Early stopping was implemented with a patience of 15 epochs, terminating training if validation performance did not improve. Additionally, the “Reduce Learning on Plateau” strategy was applied after 10 epochs, setting a minimum learning rate of 0.001 and a reduction factor of 0.2 to enhance convergence. For weight handling, the best weights were stored and saved to prevent degradation of training versus validation loss and to ensure optimal performance. It is worth mentioning that all the images utilized in the five pre-trained models were resized to a uniform shape of 224 × 224 × 3 pixels for consistency in terms of the input dimensions. Finally, Adam optimizer was utilized with a beta1 value of 0.9 and a beta2 value of 0.999 to obtain a faster convergence rate.

#### AI pre-trained models

##### VGG16 & VGG19

The pre-trained model “VGG” stands for The Visual Geometry Group. These models were used under the ImageNet Large Scale Visual Recognition Challenge (ILSVRC). It consisted of 16 and 19 layers respectively. Their uniform architecture involved the consistent use of 3 × 3 filters which could be suitable for capturing the minute details required in dental X-rays.

##### ResNet50

It is comprised of 50 layers and consists of a combination of convolutional layers, batch normalization, and residual blocks. In this study, the residual learning was dental fractures that may present subtle distinctions from healthy teeth, making them challenging to detect.

##### DenseNet121 and DenseNet169

They are both CNN architectures that belong to the family of “Densely Connected Convolutional Networks” (DenseNets). In the context of dental fracture detection, where the fusion of low-level features such as edges with high-level patterns is critical, this property enhances the accuracy of fracture identification.

### Model training and deployment

#### The voting system

To address the challenge of overfitting, commonly referred to as the bias/variance tradeoff, a technique known as “ensemble methods” was employed. Multiple learning algorithms were deployed to obtain better predictive performance than could be obtained from deploying a single model. For a proper voting mechanism, an odd number of models was typically set for use, e.g., 3, 5, or 7 combined. The more models used, the more computational power needs to be analyzed and the more time is consumed.

#### System architecture

**Fig. 1:**
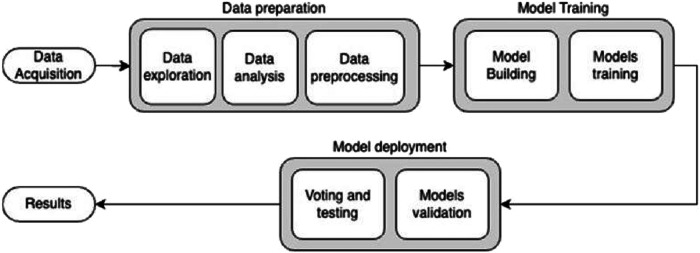
Schematic diagram for the AI-based system architecture. The proposed AI-based system is composed of three stages: First, the preparation of the dataset, then the models training stage, finally the model development stage (outcomes).

The presented diagram in Fig. 1 illustrates the sequential flow of data within the research framework. The dataset acquisition stage commenced, followed by the data preparation stage aimed at understanding the dataset and preparing it for export to the model. Subsequently, the data were subjected to stages of feature engineering and cleaning stages to enhance their quality and relevance. The first step in this phase was “Data normalization 1/255.0,” which standardized the data. This was succeeded by “Data resizing 224 × 224 × 3 pixels,” a step that ensured uniformity in the input dimensions of the data. Once these preprocessing steps were completed, the data were subsequently fed as input into the five algorithmic models for analysis, a pivotal step for predictive modeling.

The architecture displays various stages of the system. It denotes the output of each stage as an input for the following one in a schematic way. The first stage “data acquisition” was an input for the data preparation stage, and then the cleaned data was utilized in the model training stage. Finally, the voting model was deployed producing the final output result.

### Testing the diagnostic performance of the trained model

Finally, the output data from the algorithmic models were advanced to the concluding stage, characterized by the “voting classifier with a majority of voting (50% + 1 votes).” In this stage, the outputs of the models were aggregated through a voting mechanism, contributing to the final decision-making process. The diagram effectively delineates the progression of the data through these crucial stages.

Within the domain of pattern recognition and machine learning tasks such as object detection and classification, evaluating the performance of data centers on the critical metrics of precision and recall. Precision also referred to as the PPV, measures the proportion of relevant instances among the retrieved instances. The diagnostic performance of the trained model was evaluated with precision and recall. The final equation defines the PPV as a function of the true positive (TP) divided by the total number of retrieved instances of TP added to false positive (FP). Whereas in the recall function, or the sensitivity, which signifies the fraction of relevant instances that were successfully retrieved, the TP is divided by the number of total retrieved instances which included the TP added to the false negative (FN) value. Both precision and recall functions are fundamentally rooted in the concept of relevance and are widely utilized for this purpose.

## Results

### Pretrained models: VGG16 and VGG19

**Fig. 2:**
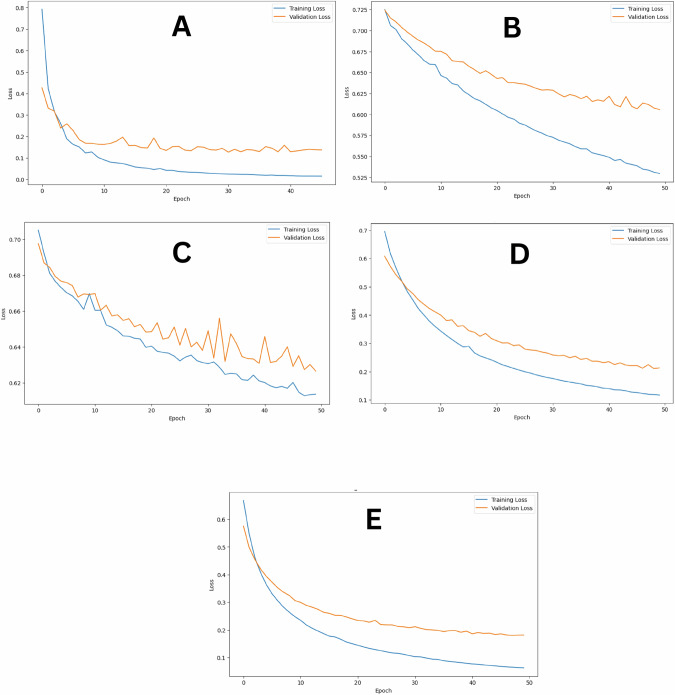
The Training loss versus validation loss for the five AI models. **A** VGG16, (**B**) VGG19, (**C**) ResNet50, (**D**) DenseNet121, and (**E**) DenseNet169. The graphs demonstrated the outperformance of VGG16, DenseNet121 and DenseNet169 over VGG19 and RensNet50. The blue line indicates the training loss, while the orange line represents the validation loss. The blue line decreases steadily over time, indicating that the model is learning and improving its performance on the training data. The closer the two curves are to each other, the better as it indicates that the network achieved better performance on the training dataset. The plotted graph suggests that the model’s performance on unseen validation data starts to deteriorate, which could be an indication of overfitting in VGG19 and ResNet50. The ResNet50 model shows a risk of overfitting compared to the VGG19 model. Better performance is noticed in both DenseNet121 and DenseNet169 models.

**Fig. 3:**
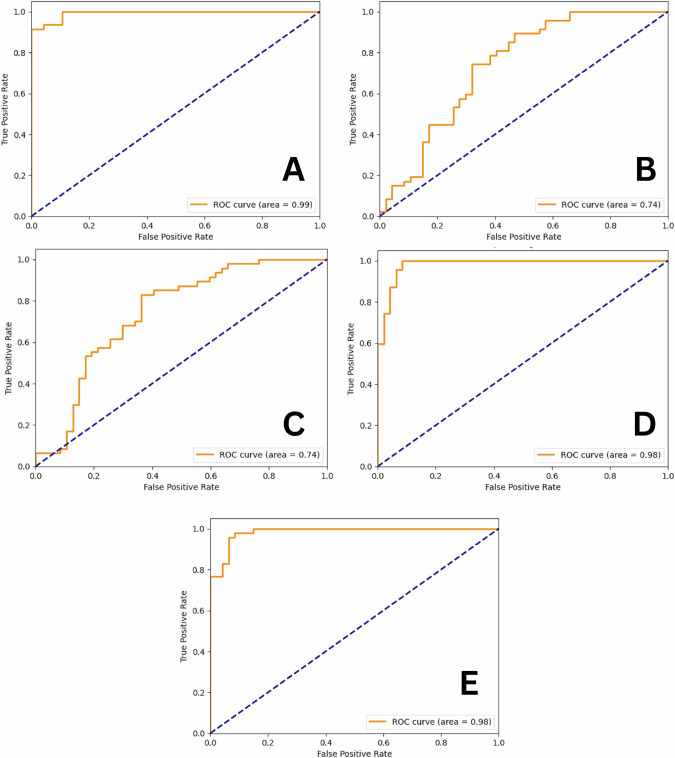
The ROC values for the five AI models. **A** VGG16, (**B**) VGG19, (**C**) ResNet50, (**D**) DenseNet121, and (**E**) DenseNet169. The curve shows the relationship between the true positive rate (TPR) on the Y axis and the false positive rate (FPR) on the X axis for different classification thresholds. A higher AUC value suggests that the model has a better ability to discriminate between fractured and unfractured teeth. The graphs demonstrated the lower performance of VGG19 and ResNet50 and the outperformance of VGG16, DenseNet121 and the ROC values approaching 1 (i.e. 100%).

The utilization of the VGG16 architecture yielded promising outcomes in the realm of dental fracture detection. The model demonstrated high performance during training with a notably low training loss of 0.09% and a validation loss of 0.18%, showcasing a strong alignment with the provided data without any apparent signs of overfitting. When assessing its ability to discern fractured teeth, the model showed a high specificity of 93.6%, ensuring accurate identification of healthy teeth. Moreover, it exhibited a sensitivity of 89.3% for the fractured class, identifying a significant portion of actual fractures. The PPV for fractures was calculated at 93.3%, emphasizing a high likelihood that the flagged teeth were genuinely fractured. In the context of nonfractured teeth, the model’s specificity reached 89.36%, and the sensitivity was 93.6%. The PPV for the nonfractured class was at 89.7%, underscoring the model’s ability to both detect and confirm the absence of fractures as shown in Fig. 2A. The receiver operating characteristic (ROC) curve’s value was 0.99 as shown in Fig. 3A. However, in VGG19, the training loss (0.55%) and validation loss (0.61%) highlighted the potential for overfitting. Notably, there was a low sensitivity of 0.447 for fractured cases, whereas a higher sensitivity of 0.745 was observed for unfractured cases. Additionally, there was a high specificity of 0.745 for fractured cases and a lower specificity of 0.447 for unfractured cases. These values translated to a predictive value (PPV) of 0.636 for fractured cases and 0.574 for unfractured cases as shown in Fig. 2B. The ROC curve’s value for VGG19 model was 0.74, underscoring the effectiveness of VGG16 compared to its deeper counterpart as shown in Fig. 3B.

### Pretrained model: ResNet50

When ResNet50 was utilized for root fracture detection, the resulting graph demonstrated that the model grappled with substantial noise, resulting in a specificity of 0.85 for the fractured class and 0.425 for the unfractured class as shown in Fig. 2C. This situation signified a model bias toward identifying unfractured cases rather than fractured cases. Additionally, this bias was evident in the ROC curve values shown in Fig. 3C, further highlighting the need for fine-tuning, and addressing imbalances to achieve a more accurate detection of both fractured and unfractured cases. This approach tackled the problem of overfitting but suffered from high noise and bias.

### Pretrained models: DenseNet121 and DenseNet169

DenseNet121 emerged as an effective solution for addressing both overfitting and noise issues in the present root fracture detection model. It achieved a well-balanced metric between training and validation losses, with values of 0.11 and 0.23, respectively. These results had a notable impact on the model’s sensitivity and specificity, which reached 0.893 and 0.936, respectively. Consequently, the PPVs for fractured and unfractured cases were 0.933 and 0.897, respectively as shown in Fig. 2D. However, the ROC curve’s value for DenseNet121 model was 0.98, confirming its effectiveness as shown in Fig. 3D.

On the other hand, for the DensNet169 model, the gap between the training and validation losses noticeably narrowed, with values of 0.112% and 0.211%, respectively. Convergence significantly enhanced the model’s generalization capability, resulting in an improved sensitivity of 0.872 and specificity of 0.936. Consequently, the PPVs increased, reaching 0.932 for fractured cases and 0.88 for unfractured cases as shown in Fig. 2E. Whereas the ROC curve’s value for DenseNet169 model was 0.98 as shown in Fig. 3E. The cumulative results of the five AI models presented as training loss, validation loss, sensitivity, specificity, PPV, and ROC values are summarized in Table 1.

**Table 1:** The collective results of the five AI models; VGG16, VGG19, ResNet50, DenseNet121, and DenseNet169.

Pretrained models	Training loss	Validation loss	Fractured roots			Unfractured roots			ROC
			Sensitivity	Specificity	PPV	Sensitivity	Specificity	PPV	
VGG16	0.09%	0.18%	0.870	1.000	1.000	1.000	0.870	0.886	0.990
VGG19	0.545%	0.61%	0.447	0.745	0.636	0.745	0.447	0.574	0.740
ResNetNet50	0.61%	0.63%	0.426	0.851	0.740	0.851	0.426	0.597	0.740
DenseNet121	0.11%	0.23%	0.894	0.936	0.933	0.936	0.894	0.898	0.980
DenseNet169	0.11%	0.21%	0.872	0.936	0.932	0.936	0.872	0.880	0.980

The data presented as training and validation loss (value in percentage), ROC value, sensitivity, specificity, and PPV values both for fractured and unfractured roots.

**Fig. 4:**
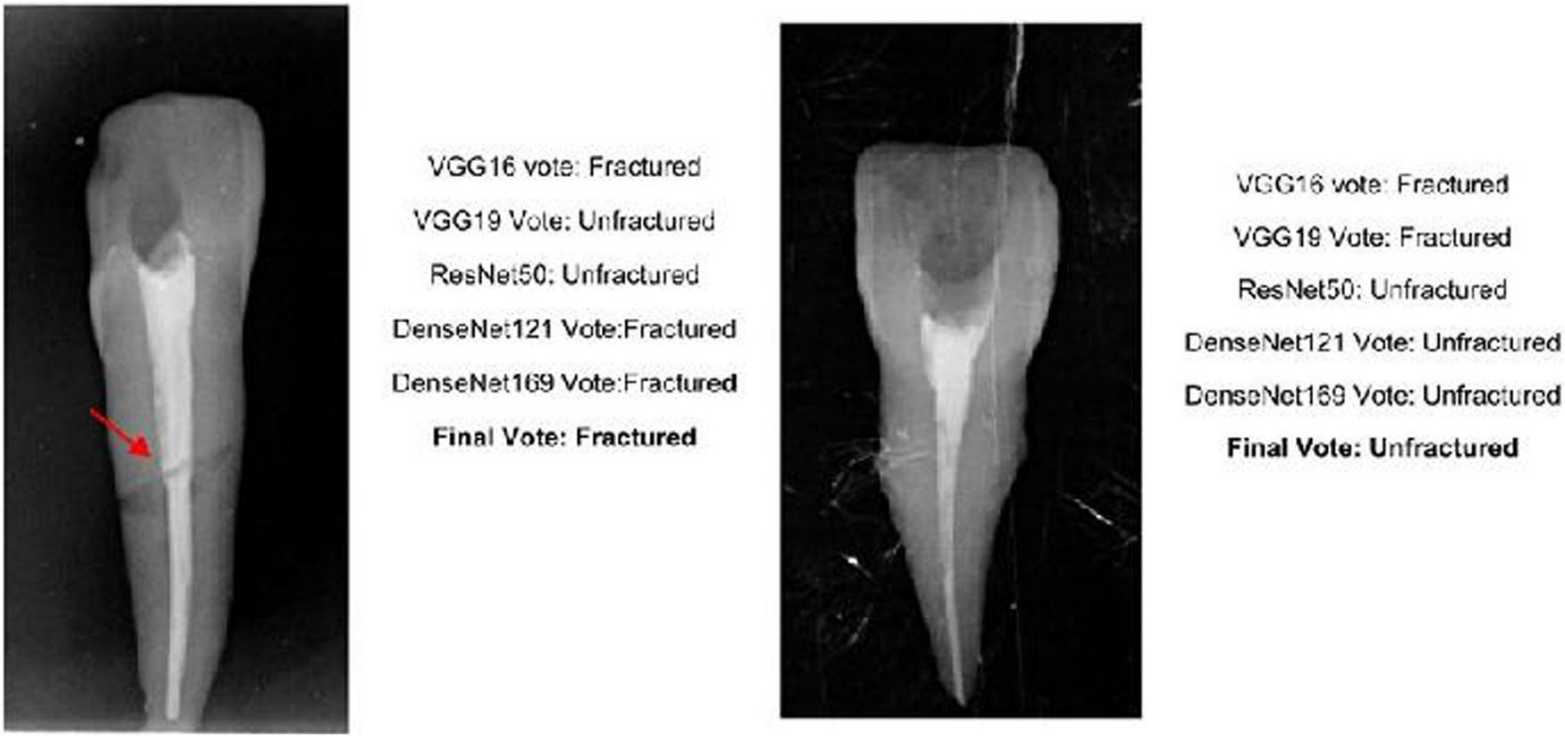
The voting system for final prediction for the 5 models. The result of each AI model for the image both for fractured and unfractured roots is given, followed by the voting decision of the AI voting mechanism. In the fractured tooth sample (on the left side), three models voted as fractured (VGG16, Dense Net121, and DenseNet169) and thus the final decision was “detected as fractured”. In the unfractured sample (on the right side), two models voted as fractured (VGG16 and VGG19) and thus the final decision was “detected as unfractured”. The arrow represents the fracture line.

The different models revealed different and inconsistent results. The voting system was implemented as explained in the system architecture to provide a concrete result for a decision in every data image as shown in Fig. 4.

## Discussion

The present study evaluated the efficiency of AI for accurately detecting dental root fracture lines in digital 2D dental periapical radiograph. This study introduces an AI-based voting system that deployed five different algorithms to overcome individual potential discrepancies between different models. Such an approach empowers low-experienced and undergraduate dentists which tackles the poor performance and reproducibility encountered in detecting root fractures [1].

Current literature supported utilizing AI in the dental healthcare system, however with particular attention to its limitations [17]. The recent literature valued the importance of ongoing developments, which can significantly impact diagnosis and decision making in dentistry [18]. Health information systems are a subsidiary of the domain of Information Systems that can significantly support in the process of diagnosis in the dental section. When using customized tools or implementing analysis, it is referred to as “Health Informatics” [19]. Using Artificial intelligence as tools to perform the analysis is commonly used in almost every intelligent system.

In the present study, five state-of-the-art AI algorithms: VGG16, VGG19, ResNet50, DenseNet121, and DenseNet169 were selected. These models were chosen for their robustness, high performance, and proven efficacy in image classification tasks. VGG16 and VGG19 are chosen for their simplicity and depth, while ResNet50 has demonstrated high accuracy in detecting vertical root fractures in CBCT scans [20]. DenseNet169 and DenseNet121 leverage dense connectivity patterns to enhance feature reuse and parameter efficiency, making them ideal choices for image analysis objectives [21, 22].

In contrast to training and validating the deep learning models on radiographic images retrieved from real world context (i.e. retrieved from patients) [23] or in ex vitro models that highly mimic it as fixing the teeth in cadaver jaws [24], unmounted extracted teeth were utilized. This choice was based on simplifying the testing model, reducing the noise produced from surrounding structures and emphasizing on accuracy of detection in consistent context. However, this ex-vivo research design does not closely correlate to the clinical settings, which encounter limitations in generalizing the results. This is due in part to the restricted availability of patient data in real-world context. Nonetheless, recent evidence revealed improved diagnostic performance when the data set was mixed with extracted teeth from in vitro model, underscoring the viability of such approach [20].

Data sets augmentations are commonly introduced to improve the generalization of the involved model. Proposed methods to introduce such variations include and are not limited to rotating, flipping, contrast adjustment and noise introduction [25]. However, such a strategy was not recruited despite the relatively small sample size for the training data sets to avoid errors like overfitting [26]. Data augmentation can retain existing biases and imbalances from the original dataset and may not consistently improve results. Moreover, inappropriate or excessive augmentation can introduce noise and artifacts, potentially degrading the model’s performance, similar to the limitations seen with synthetically produced datasets [27]. Therefore, the study would reflect more reliable results mimicking the real time context. At the heart of the methodology lies the utilization of the datasets, which were split into training and testing/validation sets, at ratio of 80:20. Adopting 80% for training provides the model with ample training data to learn patterns and generalize effectively, while reserving 20% for validation/testing allows for an objective assessment of performance, addressing overfitting and underfitting issues [28], in accordance with the best diagnostic performance and in alignment with standard machine learning practices [3, 14].

Matplotlib software, a plotting library for Python, was used in the present study to generate curves for visualization purposes of the model’s performance metrics output. Its flexibility and granular control supported in production of clear, publication-quality graphs that provided an intuitive understanding of the model’s efficacy across diverse evaluation parameters [29]. The current study employed the AI-based system for dental fracture detection based on 2D periapical radiographs of extracted teeth. Despite the limitation of two-dimensional projections, they are cost-effective and readily accessible diagnostic tools.

The present findings are in line with the previous work by Kositbowornchai et al. which was built on ex vitro model radiographic images and outlined the efficiency of neural networks for fracture detection. Although overall accuracy was varying over different parameters of training and test sample size, it could reach up to 95.7% [3].

The proven reliability of the AI-based system for root fracture detection in the current study was in accordance with the previous work of Guo et al. who incorporated the deep learning algorithm for crack detection and gained accuracy exceeding 90% in a step forward in diagnosis and decision-making automation [30]. In the former scholar, the neural network was tested on a hundred photographic optical images rather than radiographic images with a resolution of 1920 × 1080-pixel.

Noteworthy, the present results revealed the inconsistency between the five models, demonstrating varying values of PPV and ROC. Among the models, the DenseNet121 and DenseNet169 models achieved the highest sensitivity and specificity rates, making them the most effective models for both detecting fractures and identifying unfractured roots. Whereas the VGG19 and ResNet50 models showed comparable performance and were generally inferior to the other models. Moreover, ResNet50 and VGG16 demonstrated a bias towards identifying the fractured roots. The VGG16, DenseNet169, and DenseNet121 models exhibited high values approaching 100% in contrast to the rest with values ranging from as low as 42% for sensitivity of detecting fractured roots in ResNet50 model.

These findings were contradicted by Day et al. as the best performance was manifested by ResNet50 and the low performance by VGG16 [31]. This contradiction could be attributed to different methodologies which aimed for detection of dental carious in dental panoramic radiographs in contrary to the recruited-in-hand research strategy. The former study utilized the Dental Carious Detection Net (DCDNet) model with a complex architecture and Multi-Predicted Output (MPO) structure, where the AI models were deployed as blocks in “bottlenecks” structure. This resulted in varying precision and recall for different types of carious lesions where the cervical carious demonstrated the lowest values. Such discrepancies highlighted differences in system architecture and data set complexities between the two studies.

On the other hand, these results were contradicted by the results of Johari et al, where the accuracy, selectivity, and specificity were 70%, 97.7%, and 67.7% respectively in periapical radiographs [15]. This could be attributed to the differences in methodologies that aimed to validate the AI algorithms for the detection of vertical rather than horizontal root fracture, which encounter challenges in detection on periapical radiographs. Noteworthy, their employed AI model was the probabilistic neural network, trained on a significantly smaller dataset of endodontically treated teeth (120 roots), in contrast to the CNN employed in the current study, which was trained, tested and validated using a larger dataset comprising 400 root images. The discrepancies between the results of Johari et al and ours are mainly reflected in both accuracy and specificity. Moreover, the intended algorithms presented, though low, a degree of false positive prediction, which was attributed to the anatomical configurations of roots like grooves and invaginations [13].

Additionally, in contradiction to the present findings, a former study reported superiority of ResNet50 over both VGG19 and DenseNet169, where accuracy, sensitivity, and specificity were 97.8%, 97.0%, and 98.5% while 74%, 42.6%, and 85% in the current study [15]. A possible explanation is the discrepancies in methodologies, where the authors conducted a retrospective evaluation on CBCT images. The surpassing diagnostic performance of 3D image modality over digital periapical radiographs in AI-based root fracture detection model was well-proven by Johari et al. [14].

Herein, the significance of the voting system is emphasized where the voting mechanism deployed the five models for obtaining a final decision. This approach drew inspiration from the conscious voting mechanism employed by radiologists in real-world medical practice. By aggregating the insights from diverse models, model assembling leverages their collective wisdom to achieve more robust and accurate predictions, effectively addressing the challenges posed by overfitting in machine learning applications [26]. Similarly, Shrestha et al. have comprehensively examined algorithmic fairness across various domains and advocated for voting mechanisms to facilitate democratic decision-making [32].

Therefore, the combined decision is obtained by a majority vote of the individual AI classifiers. This explains the reason for having an odd number of classifiers in order to get a decisive outcome [33]. The number of the classifiers (n) could be equal to 3, 5, 7. However, the more the n, the more computing power, and the fewer the n the less reliability of the results.

The current findings agreed with the demonstrated superiority of the voting system by Shimpi et al for the detection of oral cancers [27]. Running the voting algorithm involved reflected an innovative prospective for the present study as a step forward in automation for root fracture detection.

The present study was conducted in an in vitro setting rather than a clinical environment, which limits the applicability of the findings to real-world scenarios. The use of artificially created fractures may not accurately represent the complexity and variability of fracture lines encountered in clinical practice. Future studies should focus on validating these AI models in clinical settings to ensure their effectiveness and reliability in real-world conditions. Additionally, incorporating and implementing larger data sets as well as training on images of multirooted teeth could enhance the robustness and generalizability of the AI systems. Further research should also explore the integration of these voting-based AI systems into routine dental diagnostic workflows.

## Conclusion

Within the limitations of the present study, the integration of AI-based system offers a promising approach to root fracture detection in periapical radiographs. The evaluation of the five models—VGG16, VGG19, ResNet50, DenseNet121, and DenseNet169—revealed performance discrepancies that could be addressed through a voting mechanism that enhances detection accuracy, showing potential for automated decision making.

## Data Availability

The datasets used and analyzed during the current study are available from the corresponding author on reasonable request.
